# An exfoliation and enrichment strategy results in improved transcriptional profiles when compared to matched formalin fixed samples

**DOI:** 10.1186/1472-6890-7-7

**Published:** 2007-08-03

**Authors:** Wilfrido D Mojica, Leighton Stein, Lesleyann Hawthorn

**Affiliations:** 1 Department of Pathology, University at Buffalo, The State University of New York, 100 High Street, Buffalo, NY, USA; 2 Gene Expression Facility, Roswell Park Cancer Institute, Elm and Carlton Streets, Buffalo, NY, USA

## Abstract

**Background:**

Identifying the influence formalin fixation has on RNA integrity and recovery from clinical tissue specimens is integral to determining the utility of using archival tissue blocks in future molecular studies. For clinical material, the current gold standard is unfixed tissue that has been snap frozen. Fixed and frozen tissue however, both require laser capture microdissection to select for a specific cell population to study. The recent development of a sampling method capable of obtaining a viable, enriched cell population represents an alternative option in procuring cells from clinical material for molecular research purposes. The expression profiles of cells obtained by using this procurement approach, in conjunction with the profiles from cells laser capture microdissected from frozen tissue sections, were compared to the expression profiles from formalin fixed cells to determine the influence fixation has on expression profiles in clinical material.

**Methods:**

Triplicate samples of non-neoplastic colonic epithelial cells were recovered from a hemicolectomy specimen using three different procurement methods from the same originating site: 1) an exfoliation and enrichment strategy 2) laser capture microdissection from formalin fixed tissue and 3) laser capture microdissection from frozen tissue. Parameters currently in use to assess RNA integrity were utilized to assess the quality of recovered RNA. Additionally, an expression microarray was performed on each sample to assess the influence each procurement technique had on RNA recovery and degradation.

**Results:**

The exfoliation/enrichment strategy was quantitatively and qualitatively superior to tissue that was formalin fixed. Fixation negatively influenced the expression profile of the formalin fixed group compared to both the frozen and exfoliated/enrichment groups.

**Conclusion:**

The exfoliation/enrichment technique represents a superior alternative in tissue procurement and RNA recovery relative to formalin fixed tissue. None of the deleterious effects associated with formalin fixation are encountered in the exfoliated/enriched samples because of the absence of its use in this protocol. The exfoliation/enrichment technique also represents an economical alternative that will yield comparable results to cells enriched by laser capture microdissection from frozen tissue sections.

## Background

Advances in biotechnology have made possible the elucidation of the transcriptome, defined as the complete set of RNA transcripts produced by the genome at any one time. Although most studies to date have been based on cell cultures or animal models, the value of this technology would be its application to human clinical tissue specimens [1]. Two major impediments have prevented the widespread use of expression profiling on human tissue: tissue complexity and RNA recovery and degradation. Tissue complexity refers to the presence of multiple cell types that constitute and contribute to the overall makeup of tissue. This issue is not addressed in most studies that use clinically derived tissue, although the difficulties in interpreting molecular findings from heterogeneous tissue sections has been well documented [2]. Incomplete RNA recovery and degradation are factors that can negatively impact expression profiling studies [3-5]. The most prevalent form of clinical material available for study is in the form of formalin fixed, paraffin embedded (FFPE) tissue. Issues of cell heterogeneity can now be circumvented due to the development of laser capture microdissection (LCM) [6]. However, issues relevant to obtaining a representative *in vivo *transcriptome are still present if FFPE material is the tissue source. Delays in fixation, autolysis and cross linking may alter the recovery of RNA or contribute to its degradation [7]. Therefore, studies based on FFPE tissue cannot claim to report a truly representative baseline transcriptome. Instead, most studies attempt to document the effect of FFPE on RNA integrity and transcript expression by comparing RNA related parameters between tissue fixed in different types of fixatives or tissue that has been frozen. Although frozen tissue has long been the gold standard for preservation of RNA in clinical tissue sections, to date there has been no other procurement method for comparison.

A recently developed procurement protocol capable of recovering a specific, enriched cell population from solid clinical tissue was utilized in this study to obtain fresh, unfixed cells from a resected tissue specimen [8]. The procurement protocol is capable of recovering viable cells within minutes after extirpation of a tissue specimen. The procurement technique therefore represents a close approximation of cells to their *in vivo *state, and thus can theoretically serve as the control material from which to help define a baseline transcriptome. Using this procurement technique in conjunction with analysis of frozen LCM cells, the influence of FFPE on the recovery, integrity and degradation of RNA in tissue sections can be ascertained.

The procurement technique, which comprises the action of exfoliation and takes advantage of selective enrichment, does not destroy the integrity of the underlying tissue. It therefore represents an ideal method to examine the relationship between RNA and FFPE because the area from where cells were exfoliated from can later be frozen or fixed. Using a freshly resected hemicolectomy specimen, colonic epithelial cells were obtained by one of three methods 1) exfoliation + enrichment 2) FFPE + LCM and 3) freezing of tissue + LCM. Because only one cell type from the same area was studied but collected by 3 different methods, we were able to examine the influence FFPE has on RNA recovery and integrity.

## Methods

### Tissue and cell procurement from fresh tissue

**Figure 1:**
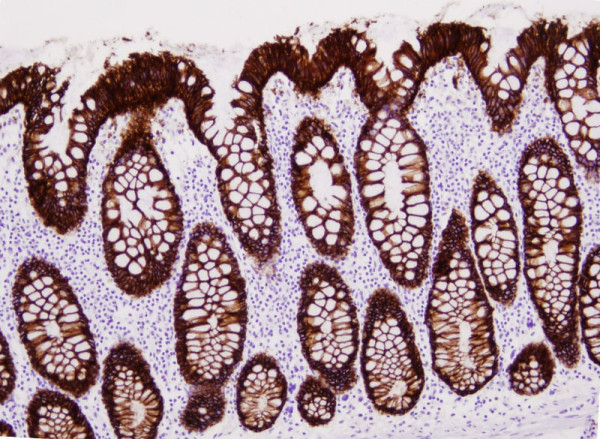
**Expression of ber-Ep4 in colon cells**. Immunohistochemistry of section of non-neoplastic colon tissue demonstrating expression by all colonic epithelial cells to the ber-Ep4 antibody. Note absence of expression in cells in the lamina propria. The difference in expression is exploited in the enrichment strategy for colonic epithelial cells from fresh tissue. (10×).

**Figure 2:**
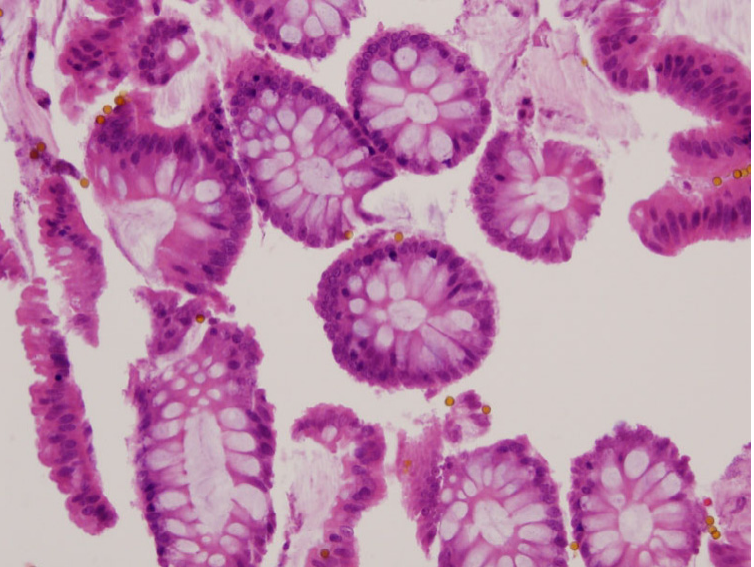
**Recovery of an enriched colon cell population using the exfoliation/enrichment strategy**. Colonic epithelial cells devoid of inflammatory cells and other cell types recovered using the exfoliation and enrichment strategy on fresh tissue. The yellow dots are the immunomagentic beads (H&E, 20×).

**Figure 3:**
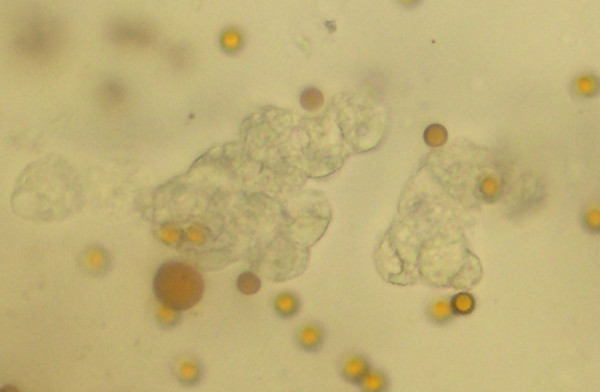
**Viability of recovered cells by the exfoliation/enrichment strategy**. The absence of blue staining in the colonic epithelial cells recovered after using exfoliation/enrichment indicates the cells recovered by this approach are viable. The yellow dots are the immunomagnetic beads (40×).

Approval for the following studies were obtained from the Institutional Review Board at the State University of New York at Buffalo. A hemicolectomy specimen excised for curative purposes was obtained in the operating suite at the time of extirpation. The specimen was transported unopened on ice to the Department of Pathology within 3 minutes where it was opened and briefly rinsed in 0.9% normal saline [9]. At a distance 5 cm from the grossly obvious tumor mass, non-neoplastic colonic epithelial cells were manually exfoliated. Manual exfoliation of the cells was accomplished by applying the edge of a glass slide to the exposed surface of the opened colonic resection specimen and gently scraping it. It is a modification of a technique initially describe by Yang who utilized it as an intraoperative diagnostic tool [10]. We quickly realized that this technique could partially purify a population of cells unencumbered by the deleterious effects of fixative. Adding laser capture microdissection to the technique enabled the recovery of highly intact nucleic acids and proteins from a select cell population [11]. We further modified our approach to expedite the entire procurement process by introducing magnetic beads bound with the antibody ber-Ep4 (Dynal Epithelial Enrich, Invitrogen) to the exfoliated cell population. This reduced the overall time frame of the procurement process and the need to purchase a laser capture microdissection machine while achieving comparable results for nucleic acid integrity and protein recovery [8]. The use of magnet beads embedded with the ber-Ep4 antibody was a logical choice because colonic epithelial cells strongly and diffusely express the epitope recognized by this antibody (Figure 1) [12,13]. The fact that no other cells in colonic tissue express ber-Ep4 allows us to selectively enrich for colonic epithelial cells (Figure 2). The enrichment step was necessary because exfoliation by itself resulted in the recovery of other cells types in the lamina propria, most notably lymphocytes and plasma cells. Enough cells were exfoliated so as to recover triplicate samples. An additional sample was collected to assess cell viability (Figure 3).

The enrichment step involved introducing the ber-Ep4 antibody bound magnetic beads to the exfoliated cells and allowing them to mix at 4°C for 20 minutes in PBS + 0.1% bovine serum albumin buffer. The unbound cells were then discarded after a magnet was used to immobilize and retain the bound cells. This wash step was performed twice.

**Figure 4:**
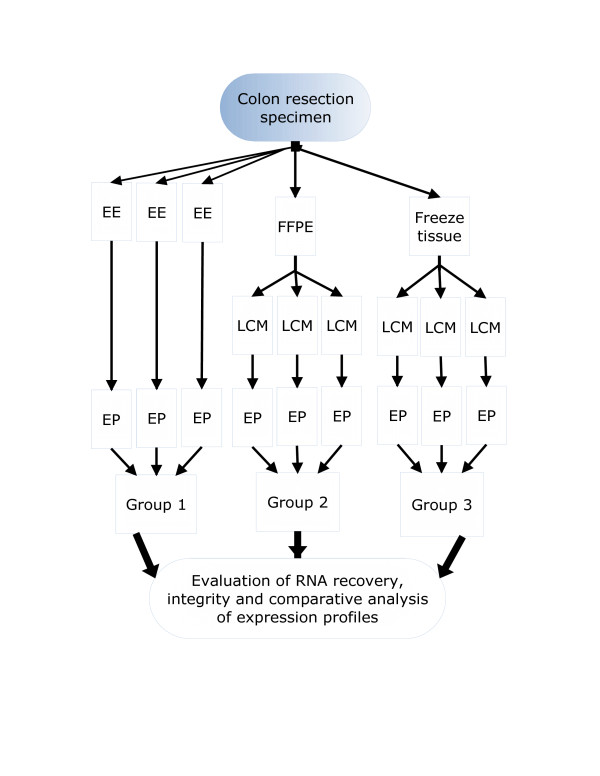
**Study design**. From a freshly resected colectomy specimen, three types of procurement were performed from tissue originating from the same site. In the fresh state, cells in Group 1 are exfoliated and enriched for using ber-Ep4(+) magnetic beads. The area from where these cells came from was then sectioned into 2 mirror pieces, with one fixed in formalin and the other frozen. For the latter two, colon cells were obtained by laser capture microdissection. After exfoliation, RNA was extracted in parallel from all the samples and afterwards underwent expression profiling on an Affymetrix microarray chip. The results were compared between the samples within each group and against the samples in other groups. EE = exfoliation/enrichment, FFPE = formalin fixed, paraffin embedded, LCM = laser capture microdissection, EP = expression profile assay.

While the colonic epithelial cells underwent the 20 minute mix with the ber-Ep4 bound magnetic beads, the colonic resection specimen was revisited. The exact section from where the cells were exfoliated was sectioned to produce two 2 cm mirror faces. We had previously found that the process of exfoliation was gentle enough that it did not destroy the tissue architecture from where the cells were procured. This added benefit therefore allowed histologic correlation of tissue with any downstream studies performed on the exfoliated cells. It is also the reasoning that formed the basis of this study. The two cut mirror faces of tissue were then excised, with one piece placed in 10% Neutral Buffered Formalin (NBF) and the other piece frozen on Optimal Cutting Media (Sakura Finatek) (Figure 4). The tissue placed in NBF was done while the exfoliated cells were undergoing the enrichment step. The tissue section was allowed to fix for 8 more hours to simulate typical fixation conditions for tissue anatomical surgical pathology specimens. Following fixation, the tissue was processing further on a Miles Scientific Tissue-Tek VIP automated processor. The program entailed an additional 2.5 hours in 10% NBF, followed by dehydration and then three separate cycles of infiltration by paraffin wax lasting 30, 60 and 60 minutes. In order to simulate archival conditions, it was maintained in storage at room temperature for one month.

The frozen section of tissue was retained in the cryostat while the exfoliated cells underwent the enrichment step. Afterwards, the tissue section was transferred to a -80°C freezer for storage.

After the two wash steps, 50 μL of a commercially available phenol+guanidine isothiocyanate solution (Trizol, Invitrogen) was added to each of the three enriched cell samples. The cells were then disrupted using a disposable minipestle (Argos Technologies). An additional 950 μL of Trizol was added to the cell lysates and cell disruption continued by repetitive pipetting. The cell lysates were then frozen and retained in a -80°C freezer. To simplify the discussion, this group will hereafter be designated as Group 1.

### Cells procured for assessment of cell viability

The sample procured for cell viability assessment was processed alongside those collected by exfoliation and enrichment. Instead of freezing and lysing the cell pellet, they were retained in PBS. Five microliters of the resuspended cell mixture was then diluted with Trypan Blue dye exclusion media to achieve a 1:20 ratio. The mixture was then transferred to a hemocytometer chamber and left undisturbed for 1 minute. The cells were then examined (Figure 3).

### Cell procurement from FFPE and frozen tissue blocks

After a month in storage, five micrometer thick sections were cut from the FFPE tissue block and placed on uncharged, RNase free slides (HistoGene™ LCM Frozen Section Staining Kit, Arcturus). The tissue section was then allowed to air dry at room temperature in a desiccant jar followed by dehydration, staining and rehydration using HistoGene™ RNase free solutions. The slides were then transferred to a desiccant jar and allowed to air dry at room temperature. In triplicate, colonic epithelial cells were laser capture microdissected using a PixCell II LCM machine (Arcturus) using a spot size of 15 μm, 70 mW of power and 295 volts. Approximately 2,000 pulses were used to procure colonic epithelial cells. This was done in triplicate. The polymer LCM cap for each sample was then fitted into a sterile microcentrifuge tube and kept on ice. Using sterile techniques, the polymer caps containing the microdissected cells were separated from the underlying caps using a scalpel blade and deposited into 3 separate microcentrifuge tubes containing 50 μL Trizol. The attached cells were disrupted as previously described before additional Trizol was added followed by freezing and retention in a -80°C freezer. The triplicate samples of cells procured by LCM from FFPE tissue sections will hereafter be referred to as Group 2.

After one month of storage, the frozen tissue block was retrieved from the -80°C freezer and allowed to equilibrate in a cryostat. The tissue block was then sectioned into 5 micrometer thick sections and placed on uncharged RNase free slides. In triplicate, colonic epithelial cells were procured by LCM in the exact same manner as previously described for the FFPE tissue block. The triplicate samples of cells procured by LCM from frozen tissue section will hereafter be referred to as Group 3.

### Extraction, purification and qualitative analysis of recovered RNA

In parallel, total RNA from Groups 1, 2 and 3 were extracted following the manufacturer's recommendations (Trizol, Invitrogen). Recovered RNA was purified using Qiagen spin columns. For RNA, values greater than 1.8, as measured by the absorbance ratio between 260 and 280 nm, were considered acceptable criteria of purity. The eluted RNA was then quantitated and analyzed through capillary electrophoresis for integrity using the RNA 6000 PicoLabChip kit on the Agilent 2100 BioAnalyzer following the manufacturer's recommendations. In addition, the raw data was analyzed using Degradometer software [14]. In this software program, RNA is categorized into one of four color annotated groups based on increasing amounts of degradation.

### Expression Microarray

Equimolar quantities of total RNA from each of the 3 groups were prepared for amplification as previously described [15]. Total RNA was isolated and cleansed using RNAeasy columns. Total RNA double stranded cDNA was synthesized using the Superscript Choice System. A T-7 (d24) primer was used to prime the first strand cDNA synthesis. An in vitro transcription reaction (IVT) was then followed by a second round of amplification. The final IVT reaction was performed in order to further amplify and biotinylate the samples. At each stage of this process the quality of the samples was monitored using both gel electrophoresis and spectrophotometry. The full length cRNAs were then fragmented to 20–200 base pairs. The quality of the array hybridization was evaluated by determining if the 5' ratio was at least half of the 3' ratio indicating that the starting poly-A RNA was of a quality to yield full-length cDNAs. We also compared the intensities of samples to each other and compared these to genes which have been spiked into the samples at known concentrations. The Affymetrix Human U133 Plus 2.0 chips used in these experiments were arrayed with sequence specific oligonucleotides representing 54,000 genes. The sequences from which the probe sets were derived were selected from GenBank^®^, db-EST, and RefSeq. The sequence clusters were created from the UniGene database (Build 133, April 20, 2001) and then refined by analysis and comparison with a number of other publicly available databases, including the Washington University EST trace repository and the University of California, Santa Cruz Golden-Path human genome database (April 2001 release). In addition, there were 9,921 new probe sets representing approximately 6,500 new genes. These gene sequences were selected from GenBank, dbEST, and RefSeq. Sequence clusters were created from the UniGene database (Build 159, January 25, 2003) and refined by analysis and comparison with a number of other publicly available databases, including University EST trace repository and the NCBI human genome assembly (Build 31). The array represents the most comprehensive coverage of the genome that is currently available. Labelled cRNA was then fragmented and hybridized unto Affymetrix HGU 133 Plus 2.0 chips.

### Analysis of microarray results

The significant differences in gene expression levels for each comparison set were derived using GC Rapid Microarray Analysis (GCRMA) software (Affymetrix). Data was normalized with a significance cut off of p = 0.05 and the false discovery rate of less than 0.05. GCRMA analyzes each hybridized chip in the context of other chips in the experiment. The algorithm consists of three steps – a sophisticated computation that uses each probe's sequence information to adjust the measured intensity for the effects of non-specific binding of G and C nucleotides in each probe, a quantile normalization stage that aligns expression values to a common distribution, and finally, an iterative median polishing procedure that summarizes the data and generates a single expression value for each probe set.

Transcript levels for the constitutively expressed housekeeping genes β-actin and GAPDH were measured as a means of assessing degradation. Measurement of the probe sets to the 3' and 5' regions to these genes can yield informative data on degradation. A three fold or greater difference in the 3'/5' ratio is one indication of degradation in an expression array data set. A numerical value of 4 or under is considered acceptable. For the remainder of genes in the transcriptome, a difference in the log 2 ratio of a gene probe set between the three groups was considered indicative of either an alteration in transcript levels or transcript loss. The loss was attributed to either degradation or poor recovery.

In order to assess the reproducibility of the procurement techniques, each sample within each group was compared to the other samples in their respective groups using Genetraffic software (Stratagene). Each scatter plot was then exported to excel to allow for the generation of Pearson correlation ratios. A linear correlation with ratios greater than 0.7 would indicate a strong positive correlation between samples collected by one procurement method. Alterations in the ratio would indicate changes occurring in the transcript levels between samples within each group. Since the samples were being compared to each other and only with those within their group, the changes in transcript levels would be attributable to either poor recovery or degradation.

### Assessing the impact of procurement method on individual gene expression profiles

Representative genes were selected from the expression profiles from each group and compared to each other. Since the study was designed so that only one cell type would be procured, the only variable different between each group would be the procurement method (Group 1 = exfoliation/enrichment; Group 2 = FFPE + LCM; Group 3 = frozen tissue + LCM). Ideally, the expression of specific genes should be similar in all three groups. Alterations in the transcript level would be attributable to the conditions associated with the procurement method. A supervised hierarchical cluster algorithm (dChip, Harvard, MA) was utilized for this comparison.

## Results

### Viability or Recovered Cells by the Exfoliation/Enrichment method

Examination of the cells dedicated for examination of cell viability by Trypan Blue staining showed that the vast majority of recovered cells excluded the dye from their cytoplasm. On average, 94% of the cells did not demonstrate cytoplasmic staining (Figure 4).

### RNA recovery and Degradometer results

**Figure 5:**
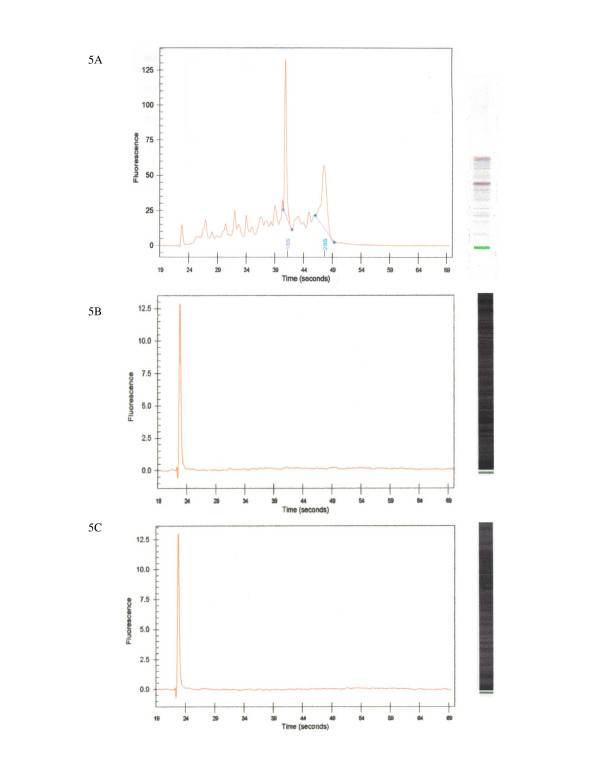
**RNA integrity**. Representative electropherograms of 18S and 28S ribosomal RNA peaks from each sample procurement group. 5A = Group 1, 5B = Group 2, 5C = Group 3. See text for details.

Sufficient RNA was recovered from every sample extraction. The average amount of total RNA from Group 1 was 1,546.8 ng/μL (Table 1). For Group 2 the average amount of total RNA was 49.27 ng/μL and for Group 3 it was 59.96 ng/μL. The average OD 260/280 absorbance ratio, a measure of nucleic acid purity, was 1.99 for Group1, and 1.69 for Group 2 and 1.70 for Group 3. Nucleic acid integrity assessed using the Agilent 2100 BioAnalyzer demonstrated the presence of 18S and 28S peaks in Group 1 but none in either Group 2 or 3 (Figure 5). The absence of 18S and 28S peaks in the latter two groups may be reflective of the enrichment process, as others have reported encountering absent ribosomal peaks but retention of intact mRNA levels for specific genes [16]. Degradometer software indicated the average degradation factor for Group 1 was 24.1%, for Group 2 it was 58.6% and for Group 3 it was 41.2%.

**Table 1:** RNA recovery for each sample group

**Procurement method**	**Total RNA (ng/μL)**	**Degradometer (range)**	**B-actin 3'/5' ratio**	**GAPDH 3'/5' ratio**
Group 1	1,546.80	Orange – red 24.1% (19.52 – 28.69)	5.6	3.8
Group 2	49.27	Black 58.6% (56.32 – 60.37)	18.4	15
Group 3	59.96	Black 41.2% (40.92 – 41.55)	9.9	19.3

Measurements of recovery and metrics of degradation for each sample procurement method for RNA in each group. Group 1 = exfoliation/enrichment approach, Group 2 = FFPE + LCM method, Group 3 = Frozen tissue section + LCM. Classification of Degradometer parameters: Yellow = degradation between 8–16%, orange = degradation between 16–24%, red = degradation > 24% and black = extensive degradation from which non-reliable data may be generated.

### Microarray results

Following 2 rounds of amplification, all samples contained sufficient RNA for the microarray hybridization. The 3'/5' ratio for β-actin for Group 1 was 5.6, 18.4 for Group 2 and 9.9 for Group 3. The 3'/5' ratio for GAPDH for Groups 1, 2 and 3 were 3.8, 15 and 19.3, respectively. The comparison between the hybridization signals between Group 1 and Group 2 demonstrated 13,544 gene probe sets exhibiting a log 2 fold change or 2 or greater between the two groups (Table 2). This represented 25% of the total gene probe sets in the chip. In the comparison between the hybridization signals between Groups 1 and 3 there were 699 gene probe sets demonstrating a log 2 fold change or greater between them. This represented 1.9% of the gene probe sets in the chip. In order to gain further insight into these changes, we examined the Gene Ontology Biologic Process ascribed to these altered gene transcripts. The largest contingents belonged to genes involved in transcription, signal transduction, protein metabolism, cell adhesion and cytoskeletal organization. In comparing the hybridization signals between Group 2 and Group 3, there were 10,344 gene probe sets showing a log 2 fold change or greater between these two groups. This represented a difference in 19% of the gene probe sets in the chip.

**Table 2:** Comparison of expression profiles between the three procurement groups

**Comparison group**	**Number of gene probe sets with ≥ 2 fold change**	**Percentage of gene probe sets with ≥ 2 fold change**
Group 1 vs. Group 2	13,544	25%
Group 1 vs. Group 3	699	1.9%
Group 2 vs. Group 3	10,344	19%

Comparison of results between sample procurement groups after amplification and hybridization onto Affymetrix HGU133 Plus 2.0 chips. Similarities in the expression profile were greatest between Groups 1 and 3, with significant differences apparent between Groups 1 and 2 and Groups 2 and 3.

### Reproducibility in procurement method

**Figure 6:**
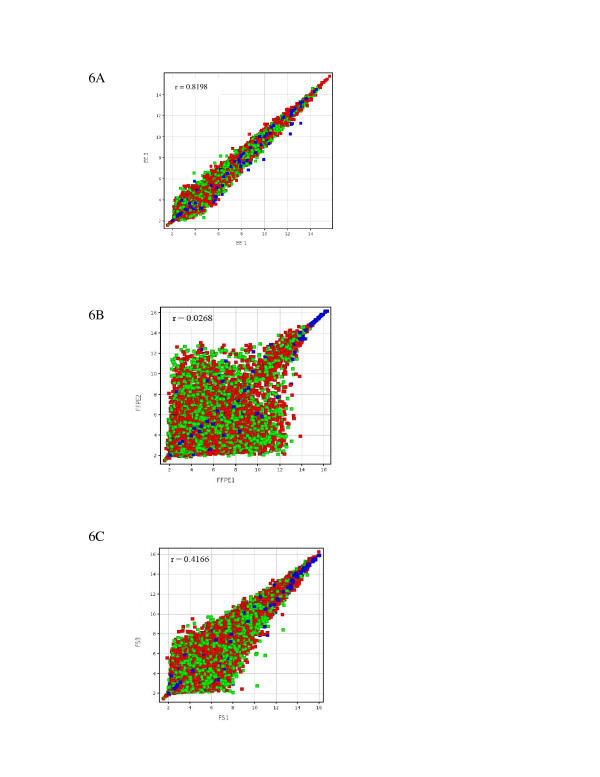
**Reproducibility of expression arrays between replicates**. Scatterplots of samples within each group to assess reproducibility of the procurement method. 6A = Representative scatterplot between two of the samples in Group 1. 6B = Representative scatterplot between two samples in Group 2. 6C = Representative scatterplot between two of the samples in Group 3.

Pearson correlation plots between like samples within each group were 0.8198 for Group 1, 0.0268 for Group 2 and 0.4166 for Group 3 (Figure 6).

### Comparative analysis of transcripts levels between matched genes collected by different procurement methods

**Figure 7:**
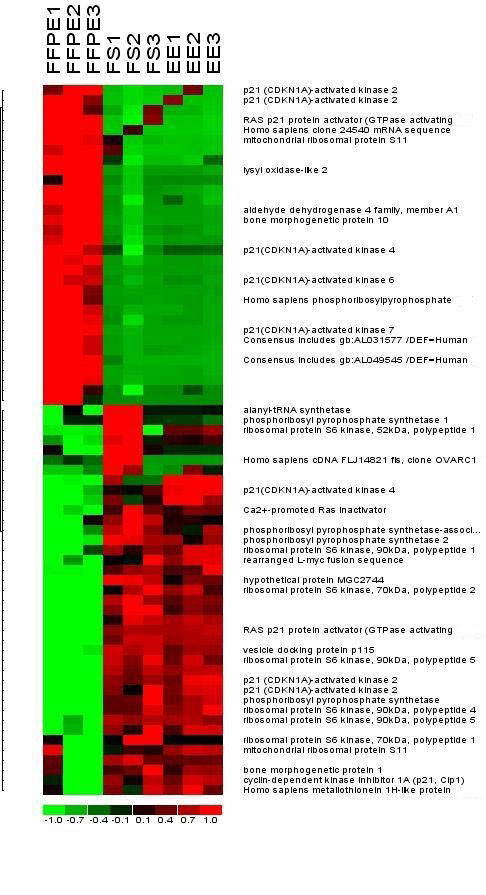
**Comparison of expression profile for selected genes between the three different procurement methods**. Expression profile of selected genes between the 3 different methods show a greater degree of similarity between Groups 1 and 3 than either with Group 2. Redundant genes indicate the presence of multiple probes, with the genes in the lower half of the cluster diagram indicating degeneration at one end of the mRNA species for that gene.

The supervised cluster algorithm demonstrated that several gene products were expressed in both Group 1 and Group 3, but were lacking in Group 2 (Figure 7). Additionally, several transcripts were found to be expressed in Group 2 but were not present in either Group 1 or Group 3.

## Discussion

A major impediment in working with clinical material is the inherent heterogeneity present in tissue specimens. The set of gene transcripts responsible for defining the homeostasis, function and phenotype for a cell are different for each cell type. Obtaining an expression profile for a portion of tissue is informative for that piece of tissue, necessitating exhaustive work to determine those transcripts specific to any cell present in the tissue. A recently developed approach to procuring a homogeneous cell population for molecular studies can be achieved through the use of laser capture microdissection. However, issues regarding the sample source introduce new variables when molecular studies are being considered. The most common and widely available tissue source is formalin fixed and paraffin-embedded. It has been repeatedly demonstrated that the process of fixation incurs alterations in the RNA molecules present in cells [3-5]. Delays in fixation, exposure to hypoxic conditions, tissue nucleases and fixative associated cross-linking are variables associated with archived fixed tissue specimens that may lead to either RNA degradation or reduced recovery [7,17]. Although there have been attempts to evaluate the effect the process of FFPE has on the expression profile of cells in clinical tissue specimens, none have compared FFPE with unfixed, enriched control cells. Trypsinizing cultured cells prior to fixation and comparing them to non-trypsinized cultured cells does not recapitulate the conditions surgical pathology specimens are exposed to [18]. Exposure to trypsin disaggregates and separates cell clusters to individual cells. Individual cells thus have a greater surface area for exposure to fixative. In contrast, cells in tissue are dependent upon the relations of the distance they are from an exposed cut surface and the speed with which formalin progresses in tissue. Thus, because most pathological tissue sections are cut at a thickness of 5 mm and formalin travels in tissue at a rate of 1 mm/hour, the transcriptome of matched cell types may demonstrate regional differences in a fixed tissue section. Although real time PCR can elucidate gene expression patterns, it is limited in the number of genes it can assess at one time. The development of newer technologies capable of assaying larger numbers of gene products holds great promise, but experiments again should be designed to replicate clinical conditions. The reproducibility or an expression profile should reflect the reproducibility of the tissue sections and not the technology [19]. In comparing the expression profiles of one section of tissue, the newly developed DASL technique demonstrated excellent reproducibility. Unfortunately, several separate tissue sections at different depths of the tissue block should have been examined to assess the effects FFPE has on reproducibility.

In this paper we introduce a technical method for the procurement of a viable, enriched cell population not adulterated by the effects of fixative. This exfoliation and enrichment approach represents a superior alternative in the procurement of specific cells relative to FFPE clinical material. This statement is based on the β-actin, GAPDH, Degradometer and ribosomal RNA (18/28S ratios) results. The one factor that is generally out of the control for procurement is pre-extirpation surgical anoxia [17]. This however, is equal in the tissue between the samples collected by the 3 different methods. Additionally, based on the amounts of RNA recovered, this procurement technique represents a more economical and expeditious method for obtaining specific cell populations from clinical material for molecular studies. A 5 ml vial of antibody bound magnetic beads costs less than $1,000. We utilized only 40 μL for each sample in Group 1. Thus, the potential to acquire 125 samples for under $1,000 exists.

The decision to examine an area of non-neoplastic colonic tissue was chosen for several reasons. First, non-neoplastic cells of the same lineage should demonstrate similar expression profiles. The heterogeneity in neoplastic clones in a tumor specimen may yield different expression profiles. Second, the method of enrichment in this technique takes advantage of the uniform and diffusely strong expression of the epitope for the ber-Ep4 antibody. This antibody, coupled to magnetic beads, enables enrichment of ber-Ep4 (+) cells once they are exfoliated from the tissue. Third, the technique of exfoliation does not destroy the underlying tissue architecture. The exact location that the cells were exfoliated from can then be excised, fixed or frozen, stained, visualized, and the exact same cell type laser capture microdissected for comparative analysis. Laser capture microdissection was necessary for the FFPE and frozen tissue sections in order that the expression profile from the same cell type could be compared to those recovered by the exfoliation and enrichment method. Finally, only one type of tissue was examined so the effects of fixative on a specific cell's expression profile could be determined. By doing each procurement method in triplicate, we could determine the reproducibility of the collection method. Group 1 demonstrated the highest Pearson correlation and most reproducible profiles, followed by Group 3. Group 2 on the other-hand, demonstrated poor reproducibility.

Since the constant between each sample group was the same cell type, and since extraction of RNA and the hybridization studies were done in parallel, the only variable present was exposure to fixative. Group 3 represents the current gold standard for expression analysis because RNA molecules are suspended by freezing for subsequent examination. Since Groups 1 and 3 were not exposed to fixative and demonstrated highly reproducible replicates, they served as a baseline from which to compare expression profiles. The expression profiles between Groups 1 and 3 demonstrated a 2 fold log 2 difference in only 1.5% of their transcripts. The differences between Groups 1 and 3 although small, may be attributable to some degree in the procurement approach of the cells in Group 1. The manipulation of these cells, exposure to buffer and binding of these cells to antibodies conjugated to magnetic beads can lead to changes in the transcript levels for certain genes. Examination of the Gene Ontology Biological Process for these gene probe sets confirmed that the majority of these changes could be attributable and the result of the manipulation the cells in Group 1 experienced. In contrast Group 2, the colonic cells that were fixed in formalin, processed to paraffin embedding and then LCM, exhibited a 25% difference in the log2 values between the genes in this group and Group 1. Examination of individual gene transcript levels between the three groups demonstrated more similarities between the two unfixed groups than with the fixed group.

Finally, the use of the exfoliation and enrichment technique may prove integral in the elucidation of the molecular events in adenocarcinoma of the colon. Currently, there exists no cell line for non-neoplastic colon cells [20,21]. In the scientific arena, alterations in transcript levels are thought to contribute to the pathogenesis of neoplasia. In the clinical arena, without an established baseline transcriptome representative of *in vivo*, non-neoplastic colonic cells, determining which genetic alterations are contributory to the pathogenesis of adenocarcinoma of the colon would be difficult. Only one type of tissue was studied so the effects of formalin fixation on RNA integrity, recovery and subsequent performance in an expression format could be examined. Because every cell type in the human body is distinctive both phenotypically and genotypically, a similar approach may need to be performed in future studies of specific cell types. In our practice, exfoliation of cells works well for the recovery of intact cells from all normal and tumor tissue types excepting bone and fat. The current limiting factor in the technique is the enrichment approach, specifically, the identification of antibodies specific to preferentially expressed plasma membrane proteins on different cell types. We envision that in the future, when the plasma membrane proteome becomes published, this technique can be expanded to include other tumor types and experiments designed to collect only specific cell types.

## Conclusion

The technique of exfoliation and enrichment enables recovery of a viable population of non-neoplastic colonic epithelial cells from clinical material. Cells recovered by this method demonstrate superior molecular parameters regarding reproducibility and integrity when compared to similar cells exposed to formalin fixation. Taking into consideration the small number of genes that have been noted to have an altered gene expression level, this technique can also be utilized as an alternative to frozen tissue.

## Competing interests

The author(s) declare that they have no competing interests.

## Authors' contributions

Each author contributed in the research performed to complete this report. WDM designed the project, obtained and performed the collection of cells by the three different procurement methods and wrote the majority of the article. LS wrote portions of the methods and results, performed the analysis of microarray data and development of the figures. LH wrote portions of the methods, results, discussion and contributed in the analysis of the data.

## Pre-publication history

The pre-publication history for this paper can be accessed here:



## References

[B1] Emmert-Buck MR, Strausberg RL, Krizman DB, Bonaldo MF, Bonner RF, Bostwick DG, Brown MR, Buetow KH, Chuaqui RF, Cole KA, Duray PH, Englert CR, Gillespie JW, Greenhut S, Grouse L, Hillier LW, Katz KS, Klausner RD, Kuznetzov V, Lash AE, Lennon G, Linehan WM, Liotta LA, Marra MA, Munson PJ, Ornstein DK, Prabhu VV, Prange C, Schuler GD, Soares MB, Tolstoshev CM, Vocke CD, Waterston RH (2000). Molecular profiling of clinical tissue specimens. J Mol Diagn.

[B2] Tureci O, Ding J, Hilton H, Bian H, Ohkawa H, Braxenthaler M, Seitz G, Raddrizzani l, Friess H, Buchler M, Sahin U, Hammer J (2003). Computational dissection of tissue contamination for identification of colon cancer specific expression profiles. FASEB J.

[B3] Benchekroun M, DeGraw J, Gao J, Sun L, Von Boguslawsky K, Leminen A, Andersson LC, Heiskala M (2004). Impact of fixative on recovery of mRNA from paraffin-embedded tissue. Diagn Mol Pathol.

[B4] Chung JY, Braunschweig T, Hewitt SM (2006). Optimization of recovery of RNA from formalin fixed, paraffin embedded tissue. Diagn Mol Pathol.

[B5] Abrahamsen HN, Steiniche T, Nexo E, Hamilton-Dutoit SJ, Sorensen BS (2003). Towards quantitative mRNA analysis in paraffin-embedded tissues using real-time reverse transcriptase-polymerase chain reaction. J Mol Diagn.

[B6] Emmert-Buck MR, Bonner RF, Smith PD, Chuaqui RF, Zhuang Z, Goldstein SR, Weiss RA, Liotta LA (1996). Laser capture microdissection. Science.

[B7] Srinivasan M, Sedmak D, Jewell S (2002). Effects of fixatives and tissue processing on the content and integrity of nucleic acids. Am J Pathol.

[B8] Mojica WD, Arshad A, Sharma S, Brooks SP (2006). Manual exfoliation plus immunomagnetic bead separation as an initial step toward translational research. Arch Pathol Lab Med.

[B9] Vincek F, Nassiri M, Knowles J, Nadji M, Morales AR (2003). Preservation of tissue RNA in normal saline. Lab Invest.

[B10] Yang GC, Hoda SA (1997). Combined use of the "Scratch and Smear" sampling technique and ultrafast Papanicolaou stain for intraoperative cytology. Acta Cytol.

[B11] Mojica WD, Rapkiewicz AV, Liotta LA, Espina V (2005). Manual exfoliation of fresh tissue obviates the need for frozen sections for molecular profiling. Cancer Cytopathol.

[B12] Gaffey MJ, Mills SE, Swanson PB, Zarbo RJ, Shah AR, Wick MR (1992). Immunoreactivity for ber-Ep4 in adenocarcinomas, adenomatoid tumors, and malignant mesotheliomas. Am J Surg Pathol.

[B13] Sheibani K, Shin SS, Kezirian J, Weiss LM (1991). Ber-Ep4 antibody as a discriminant in the differential diagnosis of malignant mesothelioma versus adenocarcinoma. Am J Surg Pathol.

[B14] Auer H, Lyianarachchi S, Newsom D, Klisovic MI, Marcucci G, Kornacker K (2003). Chipping away at the chip bias: RNA degradation in microarray analysis. Nat Genet.

[B15] Hawthorn L, Stein L, Panzarella J, Loewen GM, Baumann H (2006). Characterization of cell-type specific profiles in tissues and isolated cells from squamous cell carcinomas of the lung. Lung Cancer.

[B16] Copois V, Bret C, Bibeau F, Brouillet JP, del Rio M, Berthe ML, Maudelonde T, Boulle N (2003). Assessment of RNA quality extracted from laser-captured tissues using miniaturized capillary electrophoresis. Lab Invest.

[B17] Huang J, Qi R, Quakenbush J, Dauway E, Lazaridis E, Yeatman T (2001). Effects of ischemia on gene expression. J Surg Res.

[B18] Scicchitano MS, Dalmas DA, Beritiaux MA, Anderson SM, Turner LR, Thomas RA, Mirable R, Boyce RW (2006). Preliminary comparison of quantity, quality, and microarray performance of RNA extracted from formalin-fixed, paraffin-embedded, and unfixed frozen tissue samples. J Histo Cytochem.

[B19] Haller AC, Kanakapalli D, Walter R, Alhasan S, Eliason JF, Everson RB (2006). Transcriptional profiling of degraded RNA in cryopreserved and fixed tissue samples obtained at autopsy. BMC Clin Pathol.

[B20] Augenlicht LH, Velcich A, Klampfer L, Huang J, Corner G, Aranes M, Laboisse C, Rigas B, Lipkin M, Yang K, Shi Q, Lesser M, Heerdt B, Arango D, Yang WC, Wilson A, Mariadason JM (2003). Application of gene expression profiling to colon cell maturation, transformation and chemoprevention. J Nutr.

[B21] Mariadason JM, Arango D, Corner GA, Aranes MJ, Hotchkiss KA, Yang W, Augenlicht LH (2002). A gene expression profile that defines colon cell maturation in vitro. Cancer Res.

